# Microencapsulation of *β*-Carotene by Spray Drying: Effect of Wall Material Concentration and Drying Inlet Temperature

**DOI:** 10.1155/2019/8914852

**Published:** 2019-01-22

**Authors:** Luiz C. Corrêa-Filho, Maria M. Lourenço, Margarida Moldão-Martins, Vítor D. Alves

**Affiliations:** LEAF, Linking Landscape, Environment, Agriculture and Food, Instituto Superior de Agronomia, Universidade de Lisboa, Tapada da Ajuda, 1349-017 Lisboa, Portugal

## Abstract

Carotenoids are a class of natural pigments found mainly in fruits and vegetables. Among them, *β*-carotene is regarded the most potent precursor of vitamin A. However, it is susceptible to oxidation upon exposure to oxygen, light, and heat, which can result in loss of colour, antioxidant activity, and vitamin activity. Thus, the objective of this work was to study the microencapsulation process of *β*-carotene by spray drying, using arabic gum as wall material, to protect it against adverse environmental conditions. This was carried out using the response surface methodology coupled to a central composite rotatable design, evaluating simultaneously the effect of drying air inlet temperature (110-200°C) and the wall material concentration (5-35%) on the drying yield, encapsulation efficiency, loading capacity, and antioxidant activity. In addition, morphology and particles size distribution were evaluated. Scanning electron microscopy images have shown that the particles were microcapsules with a smooth surface when produced at the higher drying temperatures tested, most of them having a diameter lower than 10 *μ*m. The conditions that enabled obtaining simultaneously arabic gum microparticles with higher *β*-carotene content, higher encapsulation efficiency, and higher drying yield were a wall material concentration of 11.9% and a drying inlet temperature of 173°C. The systematic approach used for the study of *β*-carotene microencapsulation process by spray drying using arabic gum may be easily applied for other core and wall materials.

## 1. Introduction

Carotenoids, which are synthesized by fruits and vegetables, are a family of hydrophobic pigmented compounds that structurally exist as hydrocarbons (carotenes) or their oxygenated derivatives (xanthophylls). They are natural compounds responsible for yellow, orange, and red colours in many foods [1–3]. More than 700 different carotenoids have been isolated and identified from natural sources, of which about 50 become constituents of a typical human diet. Approximately 20 are present in human blood and tissues, such as *β*-carotene, *α*-carotene, lycopene, lutein, zeaxanthin, *β*-cryptoxanthin, *α*-cryptoxanthin, *γ*-carotene, neurosporene, and *ζ*-carotene [3–5].

These carotenoids have been recognised as potent antioxidants in humans that may play a role in preventing many diseases such as cancer, heart disease, Alzheimer's disease, Parkinson's disease, hypertension, and diabetes [6, 7]. In addition to that, other already well-known function of carotenoids (like *β*-carotene, *α*-carotene and cryptoxanthin) in humans is as provitamin A activity. Vitamin A is an essential nutrient for functions such as embryonic development, cell differentiation, vision, immunity, and reproduction [8, 9].

Among the provitamin A carotenoids, *β*-carotene is regarded the most potent precursor. In addition, it also has antioxidant action by scavenging oxygen radicals and reducing oxidative stress in the body. *β*-carotene is an orange carotenoid that is abundant in apricots, asparagus, carrots, spinach, broccoli, papaya, grapefruits, sweet potatoes, pumpkin, and paprika [10–13]. However, due to their highly conjugated structure, carotenoids are rather unstable to thermal and chemical oxidation and can be easily degraded when exposed to light, oxygen, and heat during food processing and storage [14, 15].

The stability of natural bioactive compounds, i.e., the preservation of their expected functional properties, could be improved using encapsulation techniques, such as spray drying, spray cooling, coacervation, extrusion, coating in fluidized bed, and polymerization [16]. Microencapsulation is described as a technique to entrap tiny particles of solids or droplets of liquids or gases in a biopolymer to result in small spheres which are called microcapsules or microparticles with diameters ranging from 1 to 1000 *μ*m. This technique could simplify the manufacture, handling, and storage of food, reducing production costs. In addition, the microencapsulated bioactives are protected against environmental conditions, thereby improving its stability [16–18].

In the food industry, spray drying is one of the oldest and most popular drying technologies used for microencapsulation of carotenoids due to its low cost, flexibility, production of good quality powder particles, rapid solubility of the capsules, and continuous operation [15, 19]. The structures formed in the encapsulation process are composed by two components, the core (bioactive compounds) and the protective matrix material. Core materials are dispersed in a polymer solution (wall material) and subsequently atomized into a hot chamber, which promotes the rapid removal of the water [18, 20]. The properties of the powdered particles (like particle size and distribution, moisture content, and thermal stability) may be affected by the type of wall material used and by the spray drying operating conditions such as the inlet and outlet temperatures, feed flow rate, inlet air flow rate, and atomization speed or pressure [21].

Several wall materials are commonly used, such as carbohydrates (modified starches, maltodextrin, pectins, sucrose, cellulose, arabic gum, agar, and carrageenan), proteins (gelatin, casein, and milk or soy protein), lipids (stearic acid and mono and diglycerides), and their mixtures [20, 22]. From these polymers, arabic gum is one of the most common wall materials used in microencapsulation, due to its excellent emulsification properties. It is a complex polysaccharide obtained from the branches of acacia trees, which is composed of approximately 2% protein and high proportion of carbohydrates (D-galactose, L-rhamnose, L-arabinose, and D-glucuronic acid) [21, 23, 24]. It is envisaged that carotenoids interact with the hydrophobic region of the arabic gum sample (hydrophobic proteins) via hydrophobic-hydrophobic interactions. In addition, arabic gum promotes low viscosity in aqueous media, no colour or smell, subtle taste, high oxidative stability, and good retention of volatiles [25].

Several studies regarding the carotenoids encapsulation process by spray drying have already been reported in the literature [26–32]. Though, there is a lack of a systematic study concerning the simultaneous effect of process parameters in the drying operation performance and on the properties of the particles obtained. As such, the aim of this work was to go further and study the encapsulation process of a model carotenoid molecule (*β*-carotene) by spray drying, using arabic gum as wall material, intending to evaluate simultaneously the effect of drying inlet temperature and the wall material concentration using the response surface methodology coupled with a central composite rotatable design. The response variables were the drying yield, encapsulation efficiency, particles loading, morphology, and size, as well as the antioxidant activity of the encapsulated *β*-carotene molecules.

## 2. Material and Methods

### 2.1. Materials

*β*-carotene was supplied from Sigma–Aldrich (Steinheim, Germany). Arabic gum (LabChem) was used to form the protective matrix. 2,2′-Azinobis (3-ethylbenzothiazoline-6-sulphonic acid) diammonium salt (ABTS) was purchased from Sigma–Aldrich (Steinheim, Germany). 6-Hydroxy-2,5,7,8-tetramethylchroman-2-carboxylic acid (Trolox) was obtained from Acrós Organics (Geel, Belgium). Potassium persulfate (K_2_S_2_O_8_) and ethanol were purchased from Panreac AppliChem.

### 2.2. Spray Drying Process

Arabic gum was dissolved in distilled water under stirring overnight at room temperature at the concentration values indicated in Table 1. After full hydration of the polymer molecules, *β*-carotene (5%, dry basis) was added to polymer solution and the emulsion was produced by stirring with an Ultra-Turrax T25 (IKA, Germany) at 13500 rpm for 1 min at ambient temperature. A volume of 25 mL of emulsion was prepared for each experimental condition.

The resultant emulsions were fed at a rate of 3.7 mL.min^−1^ to a cocurrent spray dryer (Lab-Plant SD-05, Huddersfield, England) equipped with a 0.5 mm diameter nozzle, a drying chamber (500 mm height and 215 mm diameter), and a cyclone (300 mm height and a bottom diameter of 90 mm). The drying air flow rate was set at 47 m^3^/h. The feed solution was kept under magnetic stirring. The pressure of the compressed air set at 1.7 bar and had a maximum flow rate of 73 m^3^/h. The inlet temperature ranged between 110 and 200°C. Encapsulation of *β*-carotene with arabic gum (5–35%) was performed according to an experimental design (Table 1). The ranges of arabic gum and inlet temperature were chosen according to preliminary results. The dried powders obtained were collected and stored under vacuum and protected from light.

### 2.3. Experimental Design

Response surface methodology (RSM) coupled with a central composite rotatable design (CCRD) was used to evaluate the effects of arabic gum concentration (5-35%) and drying inlet temperature (110-200°C) on the response variables: drying yield (DY), encapsulation efficiency (EE), morphology of microparticles, antioxidant activity of the encapsulated *β*-carotene (AA), and microparticles *β*-carotene content (LC). A total of 11 experiments were carried out (Table 1): 4 factorial design points (± 1); 4 star points (±1.414); and 3 central points (0). The repetition of the central point is used to determine the experimental error, which is assumed to be constant along the experimental domain. The experiments were performed randomly in order to avoid systematic errors.

The responses data was fitted to second-order polynomial models, using decoded variables, as follows:

(1) $$\begin{array}{c} Y_{i}=b_{0}+b_{1}X_{1}+b_{2}X_{2}+b_{11}X_{1}^{2}+b_{22}X_{2}^{2}+b_{12}{X_{1}X}_{2} \end{array}$$

where Y_i_ corresponds to the response variables; X_1_ and X_2_ represent the coded independent variables (arabic gum concentration and drying inlet temperature, respectively); b_0_ is the interception; b_i_, b_j_, b_ij_  (i, j = 1,2) are the linear, quadratic, and interaction coefficients, respectively. The adequacy of the model to the experimental data was verified by applying the analysis of variance (ANOVA) and coefficient of determination (R^2^) and adjusted R^2^ (R^2^ adj) [33]. The statistical analysis was carried out using the software “StatisticTM” version 7 (Statsoft, USA).

The optimum conditions for the microencapsulation of *β*-carotene were determined considering the results of the response variables that were significantly affected by spray drying conditions using the desirability function.

### 2.4. Antioxidant Activity before Microencapsulation

The total antioxidant activity of samples was performed by radical scavenging activity assessment expressed as Trolox Equivalent Antioxidant Activity (TEAC) described by do SM Rufino, Alves [34], and Nenadis, Wang [35] with slight modifications. An ABTS stock solution was prepared by dissolving ABTS in water at a 7 mM concentration. ABTS^+^ solution was produced by reaction of 5 mL of ABTS stock solution and 88 *μ*L of a 140 mM potassium persulfate (K_2_S_2_O_8_) solution to give a final concentration of 2.45 mM. This solution was kept in a dark room at room temperature for 12-16 h. Before analysis, ABTS^+^ solution was diluted with ethanol to obtain an initial absorbance value of 0.70 ±0.05 at 734 nm.

For the evaluation of the antioxidant activity of *β*-carotene itself, a volume of 30 *μ*L of diluted *β*-carotene with ethanol was mixed with 3000 *μ*L of ABTS^+^ solution, followed by incubation for 6 min in the dark. Then, the absorbance was measured in a spectrophotometer (Unicam, UV/Vis Spectrometer – UV4) at a wavelength of 734 nm. A calibration curve was performed using Trolox as standard antioxidant, at the concentration range of 250-2000 *μ*M in ethanol. All analytical measurements were carried out in triplicate.

### 2.5. Spray Drying and Microparticles' Characterization

#### 2.5.1. Drying Yield

Drying yield (DY) was determined gravimetrically, as described by Di Battista, Constenla [36], as the ratio of the mass of microparticles collected at the end of the spray drying process and the mass of solids contained in the feed solutions.

#### 2.5.2. Morphological Characterization of Microparticles

The morphology of the particles obtained by spray drying was observed by scanning electron microscopy (SEM). The samples were coated with a mixture of gold (80%) and palladium (20%) in a vacuum chamber and analysed using a Hitachi S2400 scanning microscope operated at 15kV with different magnifications (500x to 2000x). Particles size was measured by analysing SEM images using the image processing software ImageJ (National Institute of Health, USA) [37].

#### 2.5.3. Loading Capacity and Encapsulation Efficiency

For the determination of the concentration of the *β*-carotene present in the microparticles (LC), the method described by Rocha, Fávaro-Trindade [27] was used with some modifications. A mass of 10 mg of microparticles was added to 50 ml of ethanol. The suspension was homogenized with an Ultra-Turrax T25 (IKA, Germany) at 13500 rpm during 3 min, in order to break the particles. After mixing, the suspension was placed in amber glass flasks and kept away from light for about 12 h at 5°C. Afterwards, the suspension was centrifuged (HERMLE Labortechnik Z 383 K) at 10000 rpm during 10 min at 8°C in order to recover the supernatant. The concentration of *β*-carotene in the liquid phase (supernatant) was quantified in a spectrophotometer (Unicam, UV/Vis Spectrometer, UV4) at a wavelength of 450 nm. A calibration curve was performed with *β*-carotene diluted in ethanol with different concentrations (0.5-10 mg.L^−1^). The loading capacity (LC) of the particles was expressed as the mass of *β*-carotene per mass of particles.

The encapsulation efficiency (EE) was calculated as by Rocha, Fávaro-Trindade [27], quantifying the ratio between the mass of *β*-carotene present in the microparticles and the *β*-carotene' mass initially present in the feed solution.

#### 2.5.4. Antioxidant Activity of the Encapsulated Material

For the measurement of the antioxidant activity (AA) of the encapsulated molecules, the microparticles core materials were previously extracted with ethanol as described in the previous section. Afterwards, a volume of 800 *μ*L of supernatant was mixed with 2200 *μ*L of ABTS^+^ solution, followed by the steps described in Section 2.4 for pure *β*-carotene.

## 3. Results and Discussion

### 3.1. Microparticle Morphology and Size Distribution

**Figure 1:**
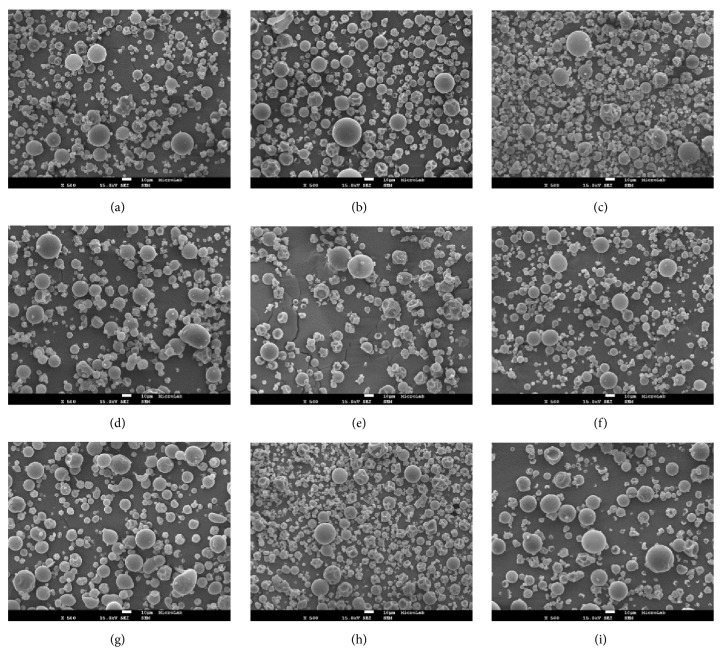
Scanning electron microscopy (SEM) images (magnification x500) of arabic gum microparticles with microencapsulated *β*-carotene. (a) 9.4 %AG, 123.2°C; (b) 9.4 %AG, 186.8°C; (c) 30.6 %AG, 123.2°C; (d) 30.6 %AG, 186.8°C; (e) 20 %AG, 155°C; (f) 5 %AG, 155°C; (g) 35 %AG; 155°C; (h) 20 %AG, 110°C; (i) 20 %AG, 200°C.

The morphological characteristics of the obtained arabic gum microparticles with *β*-carotene were investigated using SEM. SEM images have shown that the particles maintain a similar spherical-like shape with a smooth or wrinkled surface depending on the drying conditions (Figure 1).

Most of the particles did not present a significant incidence of cracks or fissures in the outer surface, indicating a resistant external physical structure. Microparticles produced without apparent damage have a lower gas permeability, presenting a more effective protection of the bioactive compounds from oxidation reactions and avoiding their undesired release [38].

Higher drying inlet temperatures tend to produce particles with a smoother surface and with a low degree of teeth and concavities (Figures 1(a), 1(b), 1(c), 1(d), 1(h), and 1(i)). This fact may be attributed to rapid water evaporation and higher pressure inside the particles during microencapsulation at higher temperatures preventing shrinking [39]. On the other hand, water diffusion is slower at lower temperatures, allowing more time for the particles to deform, wrinkle, and collapse [40]. Similar results were reported by Santiago-Adame, Medina-Torres [41] for microparticles of cinnamon infusions with maltodextrin in which the effect of three different drying temperatures (140, 160, and 180°C) was evaluated. They found microparticles morphologically more defined and smoother, without evident cracks or particle agglomerations in the spray drying process both at 160 and 180°C.

At the contrary, Figures 1(a) and 1(c); 1(b) and 1(d); and 1(e) and 1(f) show that the different values of arabic gum concentration studied did not influence substantially the particles morphology, as mixtures with a similar proportion of smooth and collapsed particles were obtained. Gonçalves, Estevinho [23] and Tonon, Brabet [42] also found no influence of the wall material concentration on the morphology of the particles obtained in the microencapsulation of vitamin A with arabic gum and açaí pulp with maltodextrin.

**Figure 2:**
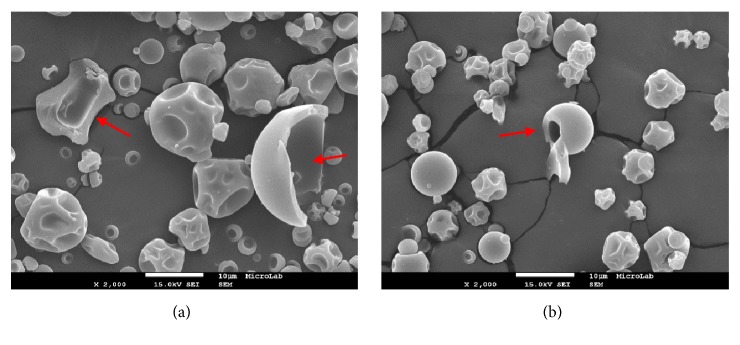
Scanning electron microscopy (SEM) images (magnification x2000) of the internal surface of Arabic gum microparticles with *β*-carotene microencapsulated. (a) 20 %AG, 110°C; (b) 9.4 %AG, 123.2°C.

The internal morphology is shown in Figure 2. All microparticles obtained were shown to be microcapsules, envisaging that the core material (*β*-carotene) was entrapped within the wall or in the centre. Central void formation, a characteristic of the spray drying process, is related to the expansion of the particles during the latter stages of the drying process, when the temperature exceeds the boiling point of the water [43, 44]. This internal structure of the microparticles was also observed in microencapsulated soybean extract microencapsulated by spray drying in arabic gum or maltodextrin matrix [45] as well as in gelatin/arabic gum microparticles loaded with fish oil [46].

Particle size distribution is a physical parameter of the powders which may influence their properties involving handling, transport, and storage such as bulk density, angle of repose, flowability, rehydration capacity, solubility, and dispersibility [17, 47]. According to Onwulata [48] and Tontul and Topuz [49] the stability of the functional components sensitive to environmental conditions is also affected by the particle size.

**Figure 3:**
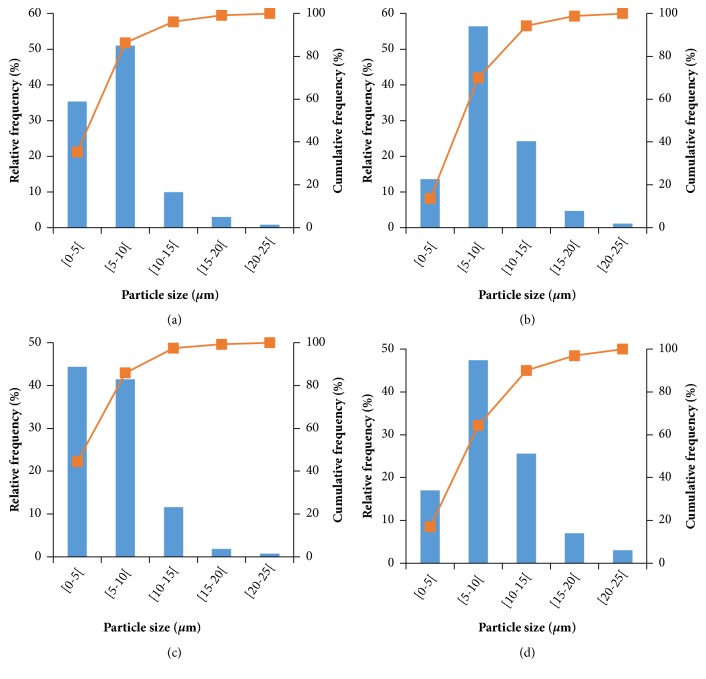
Relative frequency (bars) and cumulative frequency (line) equivalent to the diameter of microparticles. (a) 9.4 %AG, 123.2°C; (b) 9.4 %AG, 186.8°C; (c) 5 %AG, 155°C; (d) 35 %AG, 155°C.

The particle size of *β*-carotene loaded arabic gum microcapsules ranged from 1.82 to 40.91 *μ*m. In Figure 3 the particle size distribution of the microcapsules produced at different temperatures is shown (at 123.2 and 186.8°C) and with different arabic gum concentrations (5 and 35%). In all cases, more than 80% of the particles had a size below 10 *μ*m.

In general, a higher frequency of particles with sizes above 10 *μ*m was observed with increasing arabic gum concentration (Figures 3(a) and 3(b)). This fact may be related to the higher viscosity of the spray drying feed solution. According to Tontul and Topuz [49] and Tonon, Brabet [42] the liquid droplet size during atomization varies directly with the liquid viscosity at constant atomizer speed, resulting in larger particles. Similar results were obtained for different powders produced by spray drying such as blackberry juice in maltodextrin Ferrari, Germer [50] and coffee oil in arabic gum Frascareli, Silva [51].

The increase in inlet drying temperature also resulted in a higher frequency of particles with sizes above 10 *μ*m (Figures 3(c) and 3(d)). This can be related to increased swelling, thereby preventing contraction of the particle as the drying temperature increases [50, 52]. These results are in agreement with those obtained by Tonon, Brabet [42], who evaluated the microencapsulated açaí pulp in maltodextrin by spray drying. According to the authors, slower drying rate, i.e., when the inlet drying temperature is low, the particles shrink evenly, making their size smaller. However, when the drying rate is higher, the rapid evaporation of the water creates a hard crust in the particle that prevents its contraction in the drying process, resulting in larger particles.

### 3.2. Response Surface Analysis

Response surface methodology (RSM) was performed to optimize spray drying conditions, considering linear, quadratic, and interaction effects between independent variables, on the microencapsulation of *β*-carotene with arabic gum. A second-order polynomial model, described by (1), was fitted to the experimental data values obtained for each response variable studied, which are presented in Table 1. The determination coefficients (R^2^ and RAdj^2^), and the linear and quadratic effects of the factors, as well as their interaction, for each response variable are presented in Table 2.

**Figure 4:**
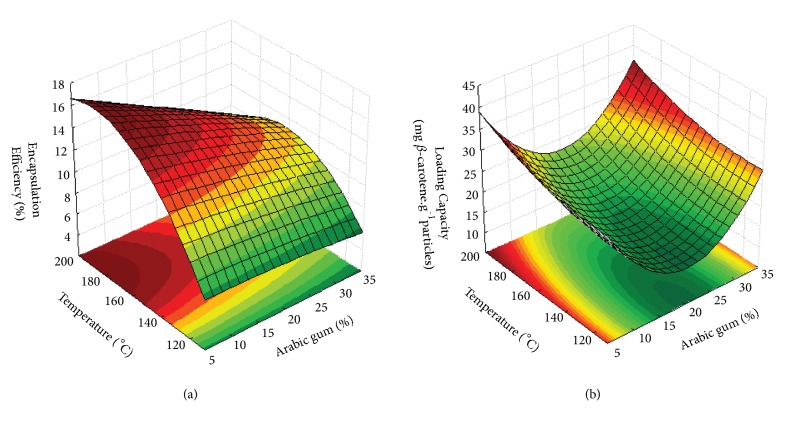
Response surface fitted to (a) encapsulation efficiency and (b) loading capacity, as a function of Arabic gum concentration and drying inlet temperature.

**Figure 5:**
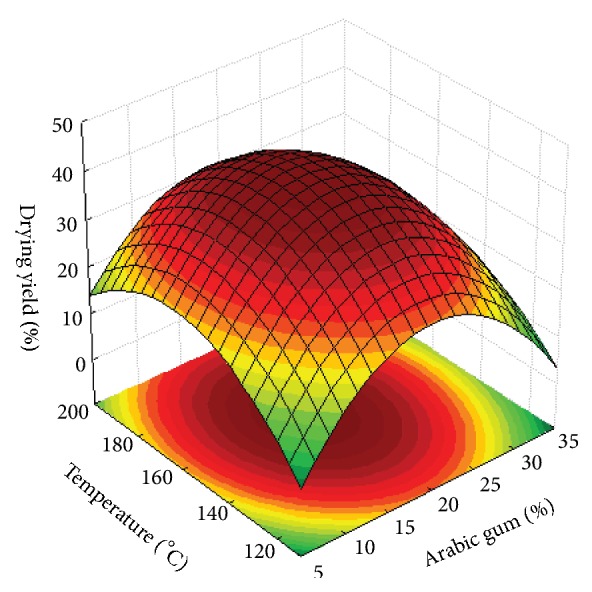
Response surface fitted to drying yield as a function of arabic gum concentration and drying.

The results show that, except for the antioxidant activity response, the mathematical model used was fitted with good determination coefficients (R^2^> 0.70). According to Lundstedt, Seifert [53], values above 0.7 represent a good fit of the model. In addition, ANOVA indicated that the lack of fit (p > 0.05) relative to pure error was not significant at 95% of confidence level. The expected errors of the models on the prediction of the responses were estimated to be 7.6%, 7.2%, and 12.7% for LC, EE, and DY, respectively. Figures 4(a), 4(b), and 5 show the 3-dimensional response surfaces that illustrate the effects of arabic gum concentration (AG; %) and drying inlet temperature (T; °C) on the responses studied.

#### 3.2.1. Encapsulation Efficiency and Loading Capacity

The encapsulation efficiency values of *β*-carotene with arabic gum ranged between 6.2 and 16.0% and the loading capacity values ranged from 11.9 to 33.6 mg*β*-carotene.g^−1^particles, as shown in Table 1. Similar results were found by Rocha, Fávaro-Trindade [27] upon microencapsulation of lycopene in modified starch that found an EE around 21% and by Botrel, Borges [54] who microencapsulated oregano oil using a mixture of arabic gum, maltodextrin and modified starch as wall material by spray drying found an EE between 5.1 and 33.9%.

As seen in Figures 4(a) and 4(b), the increase in drying temperature and decrease in arabic gum concentration lead to an increase in the EE. Regarding LC, higher values were observed at both ends of the arabic gum concentration, that is, when the lowest (5%) and the highest concentration (35%) of the wall material were used. In addition, when the drying temperature increased, the loading capacity was also increased. The same behaviour was reported by Ferrari, Germer [50] in microparticles of blackberry using maltodextrin as wall material.

According to Jafari, Assadpoor [19], the encapsulation efficiency is influenced by the drying conditions, emulsion, and bioactive compound characteristics and the wall material properties. Low encapsulation efficiency value could be due to *β*-carotene being extremely sensitive to environmental factors such as exposure to heat, light, and oxygen during encapsulation processing.

The experimental data obtained of the encapsulation efficiency and loading capacity of the particles were adjusted to the second-order polynomial model with a satisfactory coefficient of determination (Table 2). Both independent variables had a significant effect on these responses. Arabic gum concentration had a positive quadratic effect on the loading capacity of the microparticles whereas, for the encapsulation efficiency, arabic gum concentration showed a linear negative effect. In relation to the drying inlet temperature, a positive linear and quadratic negative effect on the encapsulation efficiency and a positive linear effect on the loading capacity were observed. However, the interaction coefficient was found to be nonsignificant, indicating that there was no interaction between the independent variables on the EE of particles.

The drying temperature is directly proportional to the evaporation rate and inversely proportional to the final water content of the dried microparticles. At high drying temperatures, there is a higher evaporation rate of water on the droplet surface which leads to the rapid formation of a semipermeable membrane, resulting in the protection of the release of the bioactive compounds during the drying process and, consequently, in a higher bioactive retention. However, higher drying temperatures could cause cracks and fissures on the surface of the particles leading to loss of the bioactive compound [19, 21].

Wall material concentration is also a factor that affects the retention of the bioactive compounds due to their viscosity properties in the feed solution. Some researchers have reported that the wall material concentration has a positive effect on the encapsulation efficiency; i.e., the increase of solids content in the feed increases bioactive retention [21, 55, 56]. This behaviour could be related to the reduction of the time required to form a surface crust in the atomized droplets in the initial drying process, when the solids concentration in the feed solution increases. This rapidly formed crust is not permeable to compounds, thereby protecting the bioactive from oxidation [57, 58].

However, too high viscosity of the feed solutions delays the formation of discrete particles during spray drying, whereas a low viscosity in feed delays the formation of a semipermeable surface crust, favouring further losses of the bioactive compounds [59]. Therefore, according to Reineccius [60], each wall material has its ideal feed concentration to obtain higher encapsulation efficiency, which is based on the solubility and viscosity of the feed solution. In this work, an ideal arabic gum concentration of 7.4% was found for higher values of encapsulation efficiency and loading capacity. Fernandes, Marques [57], who evaluated the effect of total solids concentration on the microencapsulation of rosemary essential oil by spray drying using maltodextrin and modified starch (1:1) as wall materials, found higher encapsulation efficiency when a concentration of the wall material of 22% was used, which was reported as the ideal concentration for maltodextrin as wall material.

#### 3.2.2. Drying Yield

Drying yield of the spray drying process is directly related to the cost of production and efficiency; thus it is an important indicator that the industry considers in its production line [49, 61]. According to Nunes and Mercadante [62] and Rutz, Borges [63], drying yield is influenced by both the equipment settings (feed rate, feed, inlet and outlet temperature, and flow rate) and drying conditions (type and wall material concentration)

In this study, a second-order model was fitted to the experimental data of the drying yield with acceptable coefficient of determination, and Table 2 shows that both independent variables, arabic gum concentration and the drying inlet temperature, had a significant negative quadratic effect on the drying yield. According to Table 1, the drying yields of *β*-carotene with arabic gum ranged between 15.9 and 43.9%. Other researchers have found values of drying yield around 50%. Roccia, Martínez [64], who studied the microencapsulation of the sunflower oil by spray drying using maltodextrin as a carrier agent, found drying yield values that ranged from 5.44 to 39.88% and Santana, Kurozawa [31] produced arabic gum microparticles with pulp pequi extract by spray drying and obtained a drying yield values between 25.8 and 56.1%.

Low drying yield in the spray drying process is mostly due to retention of the powder in the drying chamber wall, cyclone inefficient in collecting fine particles, and the high viscosity of the feed solution. This powder retention problem causes considerable economic loss and it is not cost-effective for industry as there would be frequent interruptions to the dryer cleaning, besides affecting the quality of the final product. However, drying yield in the microencapsulation technique could be improved by modifying the spray drying conditions in order to decrease the adhesion of particles to the drying chamber wall. [64–66]. According to Tontul and Topuz [49] and Jayasundera, Adhikari [67], the mechanical scraping of the drying chamber wall, introduction of cold air from the bottom, and the use of low temperature low humidity air are some examples of process-based approaches that could increase drying yield.

As shown in Figure 5, as the drying temperature and the arabic gum concentration increased, the drying yield also increased until a maximum value was achieved. After this value, decreases in the drying yield were observed even with the increase of both independent variables. The highest drying yield value was found for the sample with 20% GA dried at 155°C.

Chong and Wong [68] also found an optimum dosage of the wall material concentration (30% maltodextrin) and temperature value (180°C) that maximized the drying yield (57%), when producing sapodilla puree particles by spray drying using different maltodextrin concentrations (10-50% w/v). The authors referred that increasing the wall material concentration above the optimum value leads to an increase viscosity of the feed solution, thereby negatively affecting the drying yield.

#### 3.2.3. Antioxidant Activity

The AA values of encapsulated *β*-carotene range from 0.05 to 0.78 *μ*mol trolox.mg^−1^*β*-carotene, whereas the commercial *β*-carotene before encapsulation possessed 2.35 *μ*mol trolox.mg^−1^*β*-carotene. The lower antioxidant activity after the encapsulation may be related not only to the encapsulation process itself, but also to the incomplete extraction of the encapsulated molecules before antioxidant activity measurement. This decrease in antioxidant activity after the spray drying process was also observed, for example, by Franceschinis, Salvatori [69] in the microencapsulation of blackberry juices with maltodextrin and Hee, Tan [70] in virgin coconut oil microparticles in a mixture of maltodextrin, arabic gum, sodium caseinate, and whey protein concentrate.

The data obtained for the antioxidant activity did not fit the second-order polynomial model, though, from the results of Table 1, the two independent variables studied, arabic gum concentration and drying inlet temperature, affected antioxidant activity of the encapsulated molecules, since an AA increase was observed when the temperature decreased (runs 4-3; 2-1 and 11-10) and when the arabic gum concentration increased (runs 1-3; 2-4; 8-9).

Other researchers have also studied the influence of drying inlet temperature and wall material on AA of the particles. Kha, Nguyen [71] studied the effects of varying maltodextrin concentrations and spray drying temperatures on the antioxidant activity of Gac fruit powder and they reported that increasing the drying inlet temperature from 120 to 200°C showed a significant loss of AA. Additionally, with increasing maltodextrin concentration from 20 to 30%, the loss of AA was also observed. The authors explained that AA loss could be due to loss of antioxidant compounds present in Gac powder spray dried at high temperatures. Miravet, Alacid [72] who evaluated the antioxidant activity of pomegranate juice powder produced by spray drying using prebiotic fibers and maltodextrin as wall material also observed that the increase of the drying inlet temperature from 160 to 200°C had a significant negative effect on the antioxidant activity for both wall materials studied.

#### 3.2.4. Optimization of Drying Process Conditions

**Figure 6:**
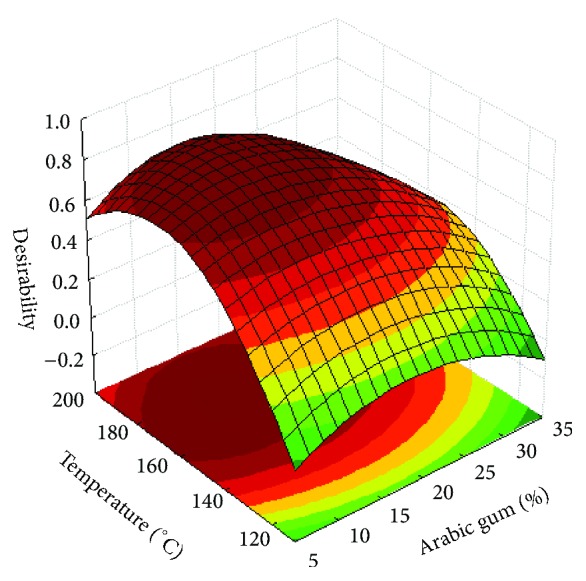
Desirability surface for optimal conditions.

The desirability function was performed for the simultaneous optimization of the responses that fitted to the second-order model (Table 2) and the desirability surface for optimal conditions is depicted in Figure 6. Desirability values higher than 0.7 were considered, indicating a good optimization of the experimental data of each response variable [73]. The best conditions for the spray drying microencapsulation of *β*-carotene with arabic gum as wall material were determined in order to obtain higher values for drying yield, encapsulation efficiency, and loading capacity.

The inlet drying temperature of 173°C and arabic gum concentration of 11.9% are recommended as the ideal conditions for microencapsulation of *β*-carotene. Under these conditions, the predicted EE, DY, and LC are 15.62%, 36.30%, and 22.74 mg *β*-carotene.g^−1^particles, respectively.

## 4. Conclusions

The microencapsulation of *β*-carotene in arabic gum by spray drying was investigated. The arabic gum concentration and drying inlet temperature influenced the drying yield, encapsulation efficiency, and load capacity responses. Regarding the AA, the antioxidant activity of *β*-carotene was reduced when microencapsulated at high temperatures (200°C) in relation to low temperatures (110°C).

SEM analysis showed that the microparticles are microcapsules. Most of them presented a similar morphology, a mixture of smooth and wrinkled particles, with a diameter lower than 10 *μ*m. Increases in drying temperature favoured the formation of smoother and larger particles.

From the experimental conditions, the drying inlet temperature of 173°C and the arabic gum concentration of 11.9% were those that allow obtaining higher *β*-carotene content, higher encapsulation efficiency, and higher drying yield.

The systematic approach used for the study of *β*-carotene microencapsulation process by spray drying may be easily applied for other core and wall materials. Further studies will focus on release studies in several aqueous media and eventually on the encapsulation of natural carotenoid extracts.

## Figures and Tables

**Table 1:** Experimental design with coded and decoded values of independent variables and spray drying responses.

Run	Independent variables		Responses variables			
	Arabic gum (%)	T	LC	EE	AA	DY
1	9.4 (-1)	123.2 (-1)	21.9	9.9	0.53	22.5
2	9.4 (-1)	186.8 (+1)	27.3	14.7	0.11	26.9
3	30.6 (+1)	123.2 (-1)	24.4	8.0	0.78	16.3
4	30.6 (+1)	186.8 (+1)	29.4	9.2	0.24	15.9
5	20 (0)	155 (0)	15.9	13.9	0.22	43.9
6	20 (0)	155 (0)	14.0	12.5	0.27	40.2
7	20 (0)	155 (0)	16.7	13.8	0.17	41.3
8	5 (-*α*)	155 (0)	33.6	16.0	0.05	23.8
9	35 (*α*)	155 (0)	26.4	12.2	0.30	23.2
10	20 (0)	110 (-*α*)	11.9	6.2	0.36	28.6
11	20 (0)	200(+*α*)	21.4	14.8	0.12	34.3

T: temperature (°C); LC: loading capacity (mg *β*-carotene.g^−1^particles); EE: encapsulation efficiency (%); AA: antioxidant activity (*μ*mol trolox.mg^−1^*β*-carotene); DY: drying yield (%).

**Table 2:** Second-order polynomial equations for each response variable.

Equation	R^2^	R_Adj_^2^
EE = −48.699*∗* + 0.3036AG − 0.001AG^2^ + 0.710T*∗* − 0.002T^2^*∗* − 0.003AG.T	0.87	0.74

DY = −175.962*∗* + 4.222AG*∗* − 0.097AG^2^*∗* + 2.245T*∗* − 0.007T^2^*∗* − 0.004AG.T	0.85	0.71

LC = 56.961–2.808AG*∗* + 0.069AG^2^*∗* − 0.257T + 0.0025T^2^*∗* − 0.0003AG.T	0.91	0.82

AG: arabic gum (%); T: temperature (°C); EE: encapsulation efficiency (%); DY: drying yield (%); LC: loading capacity (mg *β*-carotene.g^−1^particles). *∗*Affecting significantly the response variable (p>0.05).

## Data Availability

The data used to support the findings of this study are included within the article.
